# Fatal remote cerebellar hemorrhage after supratentorial unruptured aneurysm surgery in patient with previous cerebellar infarction: A case report

**DOI:** 10.1097/MD.0000000000005938

**Published:** 2017-01-27

**Authors:** Eun-Jeong Koh, Jung-Soo Park

**Affiliations:** Departments of Neurosurgery and Research Institute of Clinical Medicine, Chonbuk National University, Biomedical Research Institute of Chonbuk National University Hospital, Deokjin-gu, Jeonju, Korea.

**Keywords:** cerebrospinal fluid, remote cerebellar hemorrhage, unruptured intracranial aneurysm

## Abstract

**Rationale:**

Remote cerebellar hemorrhage (RCH) is a rare complication of supratentorial and spinal surgeries, seldom requiring intervention but occasionally causing significant morbidity or even mortality. Although a number of theories have been proposed, the exact pathophysiology of RCH remains incompletely understood.

**Patient concerns:**

We present a 62-year-old patient with RCH encountered following surgical clipping of an unruptured middle cerebral artery bifurcation aneurysm in a patient with previous cerebellar infarction.

**Lessons:**

It is extremely rare, but sometimes, RCH can be life-threatening. It is necessary to check the patient's general condition, underlying diseases and medical history. And controlled drainage of the CSF seems to be most important. Arachnoidplasty may be a consideration and the position of the drain string might have to be carefully determined.

## 1 Introduction

Most postoperative intracranial hemorrhages occur around the operative site. Remote cerebellar hemorrhage (RCH) after supratentorial craniotomy is rare, usually benign but occasionally may cause serious morbidity or mortality.^[1–6]^

Although the exact pathophysiology of RCH remains incompletely understood, there is growing consensus that the loss of a large amount of cerebrospinal fluid (CSF) may be involved.^[1–3,6]^ We report bilateral malignant RCH after surgical clipping of a middle cerebral artery (MCA) bifurcation unruptured aneurysm in a patient with previous cerebellar infarction and present a brief review of the literature.

### 1.1 Ethics statement

This study was approved by institutional review board of Chonbuk national university hospital. There is no need to obtain informed consent from the patient since all the data were collected and analyzed anonymously.

## 2 Case report

**Figure 1:**
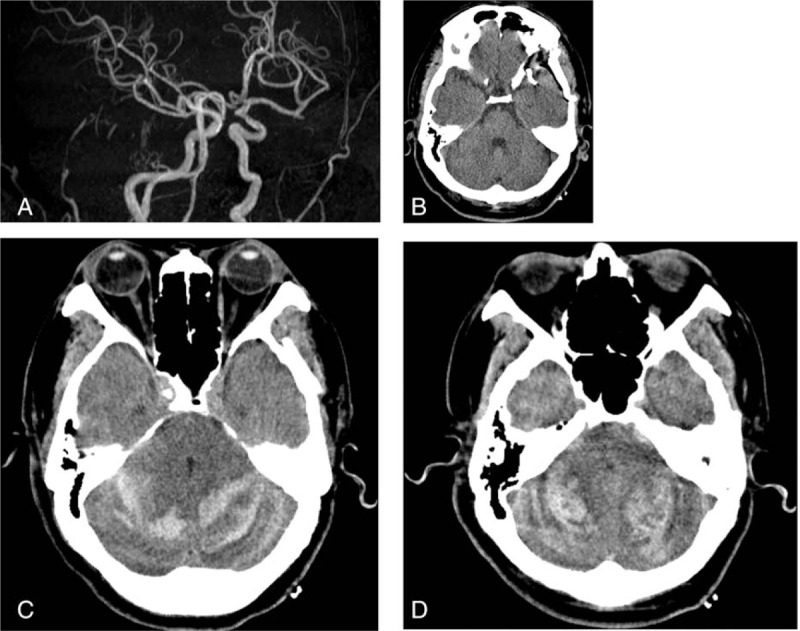
Preoperative MRA (A) shows unruptured intracranial aneurysm on left MCA bifurcation. Immediate postoperative head CT shows air density on the left frontotemporal area with a clip artifact (B). Follow-up head CT obtained 20 hours after clipping shows bilateral RCH with the characteristic zebra sign on superior folia and a massive mass effect on the fourth ventricle (C,D). CT = computed tomography, MCA = middle cerebral artery, RCH = remote cerebellar hemorrhage.

A-62-year-old male had an 4 mm unruptured left MCA bifurcation saccular aneurysm incidentally identified during work-up for a headache (Fig. 1A). He had a history of hypertension and had experienced focal left cerebellar infarction 2 years earlier. After being taken off his antiplatelet agent for 7 days, coagulation profiles of the patient were normalized. Then, a left pterional craniotomy was performed and the aneurysm was clipped uneventfully according to intraoperative neuromonitoring. The dura was closed, and the bone flap was plated. After a flat 400cc Jackson-Pratt drain was placed, the mucles and scalp were sutured anatomically. The patient's blood pressure was maintained within normal range during surgery and perioperative period. A routine immediate postoperative head computed tomography (CT) showed no abnormal findings except air density on the left frontotemporal area due to perioperative CSF drainage and the patient's neurological status was unchanged from the preoperative level (Fig. 1B). However, he developed increased sleepiness followed by agitation and confusion on the first postoperative day. Follow-up head CT scan performed 20 hours after surgery revealed bilateral RCH with a “zebra sign” (Fig. 1C and D). After 5 hours, the patient lapsed into a coma. CT scan showed additional hemorrhage with effacement of the basal cistern and a mass effect on the brain stem. Emergency posterior fossa decompression and external ventricular drainage were performed but he died 14 days later.

## 3 Discussion

RCH after neurosurgical intervention is a rare but potentially life-threatening complication. The reported incidence of RCH is around 0.08 to 0.6% of all supratentorial surgeries and 2.8% after craniotomies to clip unruptured intracranial aneurysms.^[3]^

Usually, most cases of RCH are asymptomatic and are detected on postoperative imaging. In symptomatic cases, the most common presentation of RCH is a decreased level of consciousness resulting from hydrocephalus or brainstem compression. Other common symptoms are cerebellar dysfunction, headache, motor deficits, and delayed awakening from anesthesia.^[1–8]^ Although the exact pathophygiology of RCH has not been established, it is largely accepted that it has a venous origin and is likely the result of massive CSF loss during the intraoperative and/or postoperative period.^[1–4,6,7]^ With significant CSF loss, the cerebellum may sag and stretch cerebellar bridging veins into the tentorium, torcular, and transverse sinus. These stretched veins result in transient occlusion and consequent increased venous pressure.^[1,3,6]^ The characteristic appearance of RCH on imaging, known as a zebra sign (curvilinear collections of blood paralleling the superior folia and fissure toward the tentorium), supports this theory. Moreover, the role of CSF loss is further supported by the fact that RCH tends to be more common following surgery for unruptured aneurysms compared with ruptured aneurysm.^[6]^ Generally, in the presence of acute subarachnoid hemorrhage, there is effacement of the CSF space so free CSF egress is restricted in the subarachnoid space. Besides this popular theory, various mechanisms have been suggested to contribute to RCH such as arterial hypertension, arterial capillary trauma, occult arteriovenous malformation bleeding, extreme head rotation and flexion, old parenchymal injury, coagulopathy, and drugs.

**Figure 2:**
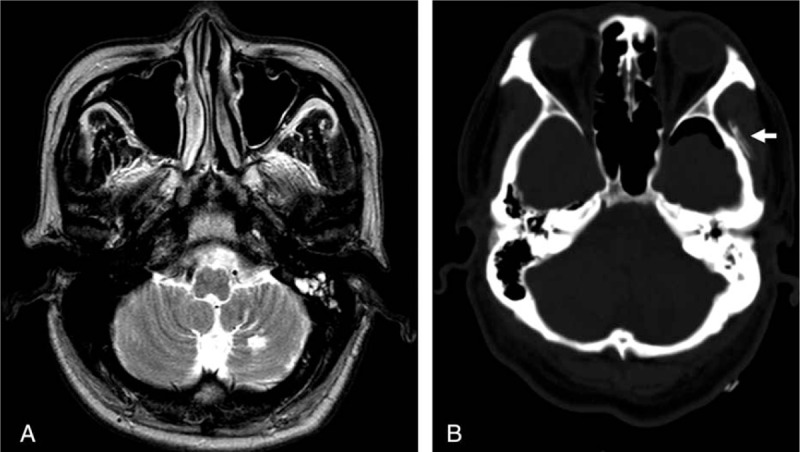
Preoperative axial T2-weighted magnetic resonance imaging shows a focal encephalomalatic change in the left cerebellar hemisphere due to previous ischemic stroke (A). Bone setting of the immediately postoperative head CT shows that 1 drain string was placed just above the temporal bone-work side (arrow) (B). CT = computed tomography.

We have presented a patient with malignant RCH after surgery for an unruptured aneurysm, who eventually died from brainstem compression. On a retrospective review of this patient, there were several points contributing to the occurrence of fulminant RCH—first, as is well known, loss of a large volume of CSF. We had opened the basal cistern for proximal control during the early stage of the operation, so CSF loss from the basal and sylvian cistern was sustained until dura closure. Second, the patient had a history of stroke and the left cerebellum showed encephalomalacia (Fig. 2A). This injury may cause damaged auto-regulation, leading to the increased blood flow and hemorrhage.^[6]^ Third, although the patient stopped taking antiplatelet agents 7 days before surgery, he had been taking aspirin for 2 years. In general, aspirin and other anti-platelet agents have been implicated as risk factors for postoperative hemorrhage. Moreover, previous studies reported that angiplatete agents or ancoagulants have been implicated as risk factors for RCH.^[1,6]^ Fourth, we think that there may have been an unexpected mistake in the later stage of surgery. We placed one 400cc Jackson–Pratt drain string just above the temporal bone-work side, which had been grinded to ensure operation visibility (Fig. 2B). This might have introduced additional negative pressure in the supratentorial space and upward traction of the tentorium. In addition to downward sagging of the cerebellum due to excessive CSF drainage, upward traction of the tentorium might aggravate the stretching of cerebellar bridging veins, such as the superior vermian vein, into the tentorium.

It is extremely rare, but sometimes, RCH can be life-threatening. Therefore, it is essential to be aware of this rare condition. Before surgery, it is necessary to check the patient's general condition, underlying diseases, and medical history. During surgery, controlled drainage of the CSF seems to be most important. Arachnoidplasty before dura closure may be a consideration and the position of the drain string might have to be carefully determined.
